# Characterizing Unsafe Sexual Behavior among Factory Workers in the Context of Rapid Industrialization in Northern Vietnam

**DOI:** 10.3390/ijerph16245085

**Published:** 2019-12-12

**Authors:** Bach Xuan Tran, Tracy Vo, Anh Kim Dang, Quang Nhat Nguyen, Giang Thu Vu, Linh Gia Vu, Khanh Nam Do, Carl A. Latkin, Cyrus S.H. Ho, Roger C.M. Ho

**Affiliations:** 1 Institute for Preventive Medicine and Public Health, Hanoi Medical University, Hanoi 100000, Vietnam; quang.n.nguyen@alumni.duke.edu (Q.N.N.); donamkhanh@hmu.edu.vn (K.N.D.); 2 Bloomberg School of Public Health, Johns Hopkins University, Baltimore, MD 21205, USA; carl.latkin@jhu.edu; 3 Department of Sociomedical Sciences, Mailman School of Public Health, Columbia University, New York, NY 10027, USA; tracy.vo@columbia.edu; 4 Institute for Global Health Innovations, Duy Tan University, Da Nang 550000, Vietnam; kimanh.ighi@gmail.com; 5 Vaccine Research Center, National Institute of Allergy and Infectious Diseases, National Institutes of Health, Bethesda, MD 20892-9806, USA; 6 UnivLyon, Université Claude Bernard Lyon 1, 69100 Villeurbanne, France; 7 Center of Excellence in Evidence-based Medicine, Nguyen Tat Thanh University, Ho Chi Minh City 700000, Vietnam; giang.coentt@gmail.com (G.T.V.); linh.coentt@gmail.com (L.G.V.); 8 Department of Psychological Medicine, National University Hospital, Singapore 119074, Singapore; cyrushosh@gmail.com; 9 Center of Excellence in Behavioral Medicine, Nguyen Tat Thanh University, Ho Chi Minh City 700000, Vietnam; 10 Department of Psychological Medicine, Yong Loo Lin School of Medicine, National University of Singapore, Singapore 119228, Singapore; 11 Institute for Health Innovation and Technology (iHealthtech), National University of Singapore, Singapore 119077, Singapore

**Keywords:** industrial worker, factory worker, sexually transmitted infections, condom use, sexual risk behavior, Vietnam

## Abstract

Industrial workers or factory workers, especially migrant workers, have been found to be vulnerable populations at risk of sexually transmitted infections (STIs). However, there has been a gap in literature regarding health behaviors of migrant factory workers. We conducted a cross-sectional study among 230 factory workers in Hanoi and Bac Ninh cities in Northern Vietnam from July to September 2018 to identify sexual risk practices and related factors among migrant and nonmigrant factory workers. Information collected regarding sexual behavior included the number of sexual partners in the previous 12 months and whether they used condoms in their last sexual intercourse. Two-thirds of participants reported having no sexual activity in the last 12 months, and there was a low percentage of participants using condoms in their last sexual intercourse. Being female, living with spouses/partners, and being a nonimmigrant had a negative association with the lack of using condoms in the last sexual intercourse with casual partners/sex workers, as opposed to having mobility and self-care problems and identifying as a binge drinker. Therefore, workplace-based prevention programs focusing on providing tailored sexual health education and promoting condom use among industrial workers, especially those who are immigrant or migrant workers, in Vietnam should be emphasized.

## 1. Introduction

The majority of migrated people in Vietnam are young adults with middle-level education status and they migrate to more urban areas mainly for employment reasons [1]. Previous studies indicated that moving to a new workplace may expose these migrant workers to new social networks, ideas, behaviors, or even socially isolated situations that could alter their lifestyle choices, including engaging in sexual risk behaviors [2]. Migrant workers are often subjected to harassment, exploitation, and violence, which may lead to poor health outcomes, and they may also face barriers to health care in the host country, such as limited or lack of health insurance and entitlement to statutory health care [3]. Migration, mobility, and family separation all relate to having a greater sense of anonymity, which might further facilitate unsafe behavioral changes, such as hazardous drinking and high-risk sexual practices [4,5]. In fact, among mobile worker populations in selected provinces in Vietnam in 2002, human immunodeficiency virus (HIV) prevalence was relatively low except among border traders in An Giang and Dong Thap provinces, which saw 2.5% and 2.1% prevalence, respectively [6]. Though this study looked at a limited group of mobile workers, it is suggested that the mobility of sex work across the border contributed to the high prevalence of HIV among border traders. In other low-middle-income countries like Vietnam, such as India and Thailand, factory workers and male migrant workers, respectively, are among the most vulnerable populations to HIV/AIDS (acquired immune deficiency syndrome) and other sexually transmitted infections (STIs) [7,8]. Migrant workers generally are at greater risk of heterosexual HIV transmission due to their demographic characteristics, such as lack of access to HIV treatment and prevention, unstable family and work situations, and access to sex workers [9].

Previous studies have found that risk perception and knowledge of consequences of unprotected sex, including HIV/AIDS, are generally low among younger migrant factory workers. For instance, in a study done in Nepal, it was reported that 90.2% of males and 41.7% of females who had nonregular sexual partners perceived having no risk of HIV/AIDS [10]. Another study conducted among migrant workers in manufacturing, construction, accommodation/catering, domestic service, wholesale/retail, and entertainment fields in China revealed that the majority of them did not use condoms while engaging in sexual intercourse, and about two-thirds of participants never or only occasionally used condoms with their casual extramarital partners within the last 12 months [11]. Studies among workers in Bangladesh, India, and Jordan also saw similar results of high levels of risky sexual practices [4,7,12]. Furthermore, a previous study conducted in Hanoi, Vietnam, found a considerable knowledge gap in HIV/AIDS prevention among male migrant workers [13]. Lack of HIV knowledge and a variety of essential health care resources were highlighted particularly among vulnerable groups, including those who were mostly low-income, employed outside of labor contracts, and unregistered for residence [13].

In the context of Vietnam, the country is rapidly transitioning into an industrial country with an increasing number of workers [14]. However, the provisions of protecting workers’ rights in using universal health coverage and accessing health services are still limited for both those who are outside labor contracts and within. Moreover, there is little evidence of literature on the high-risk sexual practices among factory workers and the underlying factors of such activities in Vietnam. Therefore, we aimed to identify the risk of sexual practices as well as related factors among factory workers in Hanoi and Bac Ninh, which are the two major cities with an increasing rate of geographic labor mobility. These findings may fill in the gap of HIV/AIDS and STI prevention of industrial workers and are intended to promote the national policy to meet the health care needs of this critical working population.

## 2. Materials and Methods

### 2.1. Study Design and Sampling

We carried out a cross-sectional study in three factories manufacturing electronics and vehicle accessories from July to September 2018 in Hanoi and Bac Ninh. In each factory, there were approximately 6000, 2000, and 1150 workers.

We used the convenient sampling technique to select participants of the study. A total of 230 workers were recruited in the study based on the following eligibility criteria: (1) Being 18 years of age and above; (2) having signed labor contracts; (3) having worked at the factory for at least 6 months; and (4) having the capacity to answer the questionnaire. Workers who were not able to communicate with the interviewers were excluded from the research. 

### 2.2. Measure and Instruments

Trained researchers conducted 20-min face-to-face interviews with participants. Briefly, we invited participants into a private working office with restricted access in order to secure their confidentiality. Study purposes, benefits, and drawbacks were clearly introduced before asking participants to join the study. Participants who agreed to take part in the study would sign written informed consent forms. 

Data collectors were researchers who had undergone research and ethics training to conduct interviews and collect data. Staff members of the factories were not allowed to participate in the data collection process. We ensured that questionnaires were anonymous by assigning participants a randomized ID number, and any information related to a participant’s identity and contact information was not collected during the research process.

A pilot survey was conducted prior to the data collection to examine the feasibility of recruitment and identify any needed modifications. There were 20 participants (both male and female with varied ages) involved in the pilot stage. The final structured questionnaire included the following information:

#### 2.2.1. Socioeconomic Characteristics

Respondents self-reported information regarding gender, age, educational level, marital status, monthly income, and whether they were living with their families. Information about immigration status, years of work experience, and the number of working hours per day was also collected.

#### 2.2.2. Sexual Behaviors

Participants were asked whether they had had sexual intercourse in the last 12 months. Those who answered “yes” were asked to report “how many partners that they had sexual intercourse with in the previous 12 months”, including intimate partners, sex workers, and casual partners (receiving and without receiving money). We also asked whether or not they used condoms in their last sexual intercourse with these partners.

#### 2.2.3. Health Risk Behaviors

Drinking pattern was examined using the Alcohol Use Disorders Identification Test-Consumption (AUDIT-C) [15]. This tool contained 3 questions with the overall score ranging from 0 to 12. The higher score of AUDIT-C suggested a greater risk of alcohol dependence. Drinking patterns were also determined. Participants were identified as hazardous drinkers if the total score of AUDIT-C was higher than 4 for males and higher than 3 for females. Binge drinkers were identified as having any positive response to the following question: “How often do you have six or more drinks on one occasion?” 

We divided the smoking status of participants into 3 types: Current smokers, former smokers, and never-smokers [16]. A current smoker was an adult who had smoked at least 100 cigarettes in his or her lifetime and had smoked in the last 30 days. The former smoker was identified as an adult who had smoked at least 100 cigarettes in his or her lifetime, but had quit smoking at the time of interview. A person who had never smoked, or who had smoked less than 100 cigarettes in his or her lifetime was defined as a never-smoker.

#### 2.2.4. Health Status

Health-related quality of life (HRQoL) was assessed by the Vietnamese version of the EQ-5D-5L questionnaire [17]. There were five health aspects that were measured, including mobility, self-care, usual activities, pain/discomfort, and anxiety and/or depression. Each domain was assessed by a Likert scale (no problems, slight problems, moderate problems, severe problems, and extreme problems). Respondents also self-reported their current acute and/or chronic conditions, the number of health problems they had had, and whether they had utilized reproductive health services in the last 12 months. 

### 2.3. Statistical Analysis

Data were analysed using STATA 12.0 (Stata Corp. LP, College Station, TX, USA). Socioeconomic characteristics, health risk behavior, and health status were compared between those using and not using a condom in the last sexual intercourse with casual partners and/or sex workers. Generalized estimating equations (GEE) binomial regression was conducted to identify the factors related to not using a condom with casual partners and/or sex workers. GEE was used to control the potential correlation of outcomes between respondents from the same factory, assuming an independent correlation structure. The robust standard errors were estimated, adjusting for the clustering within factories. A *p*-value less than 0.05 was statistically significant.

### 2.4. Ethics Approval

The research protocol was approved by the Institutional Review Board of the Hanoi Medical University (code: 01a-QD/VNCTN).

## 3. Results

Table 1 highlights the demographic characteristics, general health status, and drinking/smoking practices of the participants. The majority of the participants were female (81.2%) and about two-thirds of respondents had high school education (61%). Almost all of the participants (96.5%) had a spouse or partner. The mean monthly income was $282.40 (SD = 106.5), and the mean working time was 9.8 years (SD = 3.7). There were 16.6% and 15.7% of participants who were considered hazardous drinkers and binge drinkers, respectively. The majority of participants were never-smokers (85.5%) and had acute or chronic conditions (84.2%). Approximately half of participants had used reproductive health services (45.0%).

Table 2 shows that, among the approximately two-thirds of participants who had sex in the last 12 months (67.8%), 64.8% had one sex partner. Almost half of the respondents reported using condoms when they had sex with their spouse/intimate partners in their last sexual intercourse (42.6%). Among those who had sexual intercourse with sex workers, casual partners without receiving money, and casual partners while receiving money, 38.3%, 39.3%, and 43.9%, respectively, reported using condoms in their last sexual intercourse.

Table 3 assesses the factors related to not using condoms with sex workers and/or casual partners. Workers who were female, had completed high school education or above, or were living with a spouse/partner were less likely to not use condoms with casual partners or sex workers. By contrast, workers who were immigrants or migrants, considered to be binge drinkers and reported having mobility and self-care problems, were more likely to not use condoms in their last sexual intercourse with casual partners or sex workers. 

## 4. Discussion

This research is among the few studies that examine the sexual risk practices among factory workers in Northern Vietnam. We reported a low percentage of condom use among the workers in their last sexual intercourse with sex workers and/or casual partners. In addition, we found that being a female worker, living with spouses/partners, and being a local (and not a migrant) worker were negatively associated with no condom usage in the last sexual intercourse with casual partners or sex workers. By contrast, having problems in mobility, having self-care problems, and being a binge drinker were positively associated with a lack of condom usage in the last sexual intercourse with sex workers and/or casual partners. Our results provide evidence supporting the enhanced implementation of critical sexual health interventions for people working in industrial zones in Northern Vietnam.

In this study, we found a low percentage of participants using condoms in their last sexual intercourse with sex workers and/or casual partners. This finding is lower than the result of a previous study in India, which found that nearly 60% of workers used condoms in their last sexual intercourse with nonspousal partners [7]. However, when compared to the percentages of condom usage among migrant workers and their nonspousal partners in another study in India, our study showed higher percentages of condom usage with nonspousal, unpaid partners, but not with sex workers [18]. Another study done in China showed that returning rural-to-urban migrant workers had an elevated risk of risky sexual behaviors over nonmigrant workers [19], as our study also indicates. In spite of Vietnam’s large scale “100% condom use program”, which directly promoted the use of condoms especially in sex work as part of the National Strategy on HIV/AIDS Prevention and Control [20], our findings show that a high percentage of factory workers engaged in high-risk sexual activities. This is concerning given that unprotected sex is considered one of the main risks of infecting HIV via heterosexual intercourse [21]. Further analysis of factors underlying condom use preferences among industrial workers may provide important insight into developing more effective promotion of condom use in this high-risk population. 

Our study suggested that female workers and those who were living with spouses or partners were less likely to not use condoms in their last sexual intercourse. First, in terms of marital status, a previous study done in Chiang Mai, Thailand, showed that factory workers without a cohabiting partner were more likely to engage in higher-risk sexual activity, as compared to those with a cohabiting partner [21]. Moreover, migrant workers living alone were at greater risk of sexual behaviors potentially exposing them to HIV transmission [22,23]. Regarding the association between being a female worker and condom use, it should be noted that most of our study participants were female, which could have impacted our results [24,25]. In fact, a woman’s judgment of her own ability to negotiate and practice a given sexual behavior has been found to be a strong predictor for condom use in the last sexual intercourse with her husband [26]. Similarly, another study found that the more a female student perceived that women were subordinate to men, the lower a female student’s self-efficacy related to sexual communication, which involves communicating preference for condom use with their partners [27]. Moreover, a study conducted among factory workers in Chiang Mai, Thailand, revealed that sexual behaviors are normalized among men, but not women among Chiang Mai factory workers [21]. Lastly, a high proportion of female migrant workers in the industrial parks in Vietnam considered condoms as a contraceptive method as well as preventing sexually transmitted diseases [28].

In contrast, we found that migrant and immigrant workers had higher odds of not using condoms in the last sexual intercourse with sex workers and/or casual partners. Sexual behaviors of migrants and immigrants often changes upon moving, and the feeling of anonymity in a new region or country may increase the likelihood of participating in risky sexual activities, such as alcohol abuse, multiple sex partners, or having sexual intercourse with commercial sex workers [11,29]. Studies in the past further indicated that such high-risk sexual activities might be the social-psychological characteristics of some migrant workers. Specifically, migrant workers, who are often forced into physically demanding jobs with poor living conditions and little to no benefits while having to live apart from their spouses and families, may develop new sexual relationships and engage in high-risk behaviors that may increase the probability of HIV infection [30,31]. For example, Chen et al. emphasized the relationship between alcohol use risk and a series of sexual risk behaviors (early sexual debut, not utilizing condoms, and having sex under the impact of alcohol) in China, but saw this among female sex workers [32]. Furthermore, as found in our study, the co-occurrence of alcohol use and unprotected sex has been reported in other settings [33,34,35,36]. Aside from sexual risk behaviors and other risky behaviors, migration status remained significant even when we accounted for certain mental health status indicators, such as anxiety and depression and having self-care problems. This suggests that factors related to a worker’s identity as a migrant or immigrant affects condom use and other unsafe sexual behaviors.

In addition, health status was another factor we saw associated with low condom use. Agreeing with previous findings [37], we found that those who reported having mobility issues or self-care problems had higher odds of not using condoms with casual partners and/or sex workers. Our study’s results with health status indicators were similar to those of Nguyen et al. where drug use and sexual risk behavior among male laborers were associated with psychological factors, social integration, and social barriers [13]. Having physical and mental health problems, in fact, have been found to be associated with unsafe sexual activity [38,39], which may cause psychosocial and cognitive impairment and reduce one’s capacity to avoid engaging in risky behaviors [40].

Our findings emphasized the importance of implementing workplace-based health programs that focus on providing tailored education to prevent risky sexual behaviors. These programs should be free and targeted to promote condom use among industrial workers in Vietnam. As the majority of workers are low-wage and have limited formal education, these prevention programs should keep in mind the health literacy of workers and should approach workers and their families through broader public health messages. Additionally, an enhanced alcohol abuse screening program may help facilitate safe sex practices among workers. Such programs should be targeted at populations that are at higher risk of engaging in unprotected sexual activities, such as single male workers, migrant workers, and workers with mobility issues and/or self-care problems. A preliminary study evaluated the behavioral outcomes of an HIV counseling and testing service that integrated sexual and reproductive health services for young people living in industrial zones in Vietnam [41]. This study found that willingness to obtain an HIV test increased significantly and saw changes towards more positive knowledge, attitudes, and risk perception towards HIV/AIDS [41]. Results from our study also provided critical evidence for assessing sexual health risk behavior of industrial workers, which may help to promote national policies to meet the health care needs of this critical working population. 

It should also be noted that our study has several limitations. First, the cross-sectional design constrained our ability to establish causal relationships regarding the associated factors and sexual risk behaviors. Second, convenience sampling may have reduced the generalizability of the study results to a larger factory worker population. In addition, our sample size of 230 factory workers from three different factories in Northern Vietnam may not have fully represented all factory workers in the country. Finally, self-reporting sensitive behaviors, such as alcohol use, self-care issues, mental illness, and risky sexual habits, may have been underreported due to the participants’ social desirability and recall bias. These behaviors may be underreported due to a worker’s fear of job loss or potential consequences about one’s behaviors, despite this study’s confidentiality and anonymity.

## 5. Conclusions

Our findings highlight the high percentage of workers in the sampled factories in Northern Vietnam who did not use condoms during their last sexual intercourse with sex workers and/or casual partners. Several characteristics that were associated with such risky sexual behavior included being immigrant and migrant workers, identifying as a binge drinker, and having mobility and self-care problems. Future workplace-based sexually transmitted infection prevention programs should focus on enhanced sexual education and promotion of safe sex practices among these high-risk populations.

## Figures and Tables

**Table 1:** Demographic characteristics, general health status, and behaviors of participants (n = 230).

Characteristics	n	%
**Gender (Female) (n = 230)**	186	81.2
**Education attainment (n = 228)**		
Under high school	17	7.5
High school	139	61.0
Above high school	72	31.5
**Marital status (n = 230)**		
Single	8	3.5
Having spouse/partner	222	96.5
**Currently living with (n = 228)**		
Parents	115	50.4
Wife/husband	187	82.0
Children	139	61.0
Brothers/sisters	18	7.9
Relatives	2	0.9
Colleague	2	0.9
**Immigrants/migrant workers (n = 229)**	117	51.1
**Having problems in (n = 230)**		
Mobility	76	34.1
Self-care	7	3.2
Usual activities	73	32.7
Pain/Discomfort	132	61.4
Anxiety/Depression	135	62.8
**Having acute and/or chronic conditions (n = 222)**	187	84.2
**Using reproductive health service in the last 12 months (n = 220)**	99	45.0
**Hazardous drinking (n = 223)**	37	16.6
**Binge drinking (n = 223)**	35	15.7
**Current smoking status (n = 214)**		
Never-smokers	183	85.5
Former smokers	14	6.6
Current smokers	17	7.9
	**Mean**	**SD**
**Age**	31.7	4.5
**Monthly income (USD)**	282.4	106.5
**Years of experience**	9.8	3.7
**Working hour per day**	8.3	0.9
**Number of acute and/or chronic conditions**	1.8	1.7

**Table 2:** Sexual behaviors in the last 12 months among participants.

Characteristic	Local People (n = 113)		Migrants (n = 117)		Total		*p*-value
	n	%	n	%	n	%	
**Having sex (n = 224 )**	71	64.6	77	67.5	148	66.1	0.80
**Number of sex partners (all types) (n = 225)**							
None	20	18.2	17	14.9	38	16.5	0.83
One sex partner	69	62.7	76	66.7	145	64.8	
Two sex partners or more	2	1.8	1	0.9	3	1.3	
Don’t remember	19	17.3	20	17.5	39	17.4	
**Using condom in the last sexual intercourse with**							
Spouse/intimate partners (n = 214)	47	46.5	49	43.4	96	44.9	0.64
Yes	47	46.5	49	43.4	96	44.9	0.64
No	54	53.5	64	56.6	118	55.1	
Sex workers (n = 177)							
Yes	11	12.8	7	7.7	18	10.2	0.15
No	16	18.6	10	11.0	26	14.7	
Do not remember	59	68.6	74	81.3	133	75.1	
Casual partners without receiving money (n = 187)							
Yes	12	13.5	8	8.2	20	10.7	0.44
No	16	18.0	16	16.3	32	17.1	
Do not remember	61	68.5	74	75.5	135	72.2	
Casual partners while receiving money (n = 178)							
Yes	11	12.9	6	6.5	17	9.5	0.23
No	11	12.9	9	9.7	20	11.2	
Do not remember	63	74.1	78	83.9	141	79.2	

**Table 3:** Factors associated with not using a condom with casual partners or sex workers (n = 208).

Characteristics	Not Using Condom with Casual Partners/Sex Workers in the Last Sexual Intercourse		
	OR	95% CI	*p*-value
**Gender (Female vs. Male)**	0.52 ***	0.34; 0.79	0.00
**Education (vs. Less than high school)**			
High school	0.12 ***	0.03; 0.43	0.00
Above high school	0.14 **	0.02; 0.98	0.05
**Marital status (Living with spouse/partner vs. Single)**	0.19 **	0.04; 0.96	0.05
**Immigrants/Migrants (Yes vs. No)**	2.49 ***	1.60; 3.88	0.00
**Age**	1.06 **	1.01; 1.11	0.03
**Having problems in**			
Mobility problems (Yes vs. No)	2.84 ***	1.36; 5.93	0.01
Self-care problems (Yes vs. No)	4.85 ***	2.48; 9.47	0.00
Usual activities problems (Yes vs. No)	0.52 *	0.26; 1.04	0.06
Pain/Discomfort (Yes vs. No)	2.13	0.26; 17.56	0.48
Anxiety/Depression (Yes vs. No)	0.93	0.42; 2.10	0.87
**Having acute or chronic conditions (Yes vs. No)**	0.94 ***	0.89; 0.98	0.01
**Using reproductive health service in last 12 months (Yes vs. No)**	1.47	0.57; 3.77	0.42
**Hazardous drinking (Yes vs. No)**	0.30	0.04; 2.09	0.23
**Binge drinking (Yes vs. No)**	3.97 **	1.12; 4.15	0.03
**Current smoking status (vs. Never-smokers)**			
Former smokers	1.06	0.54; 2.09	0.87
Current smokers	0.37 **	0.15; 0.91	0.03

*** *p* < 0.01, ** *p* < 0.05, * *p* < 0.1.
